# HLA Class Ib Molecules and Immune Cells in Pregnancy and Preeclampsia

**DOI:** 10.3389/fimmu.2014.00652

**Published:** 2014-12-23

**Authors:** Snezana Djurisic, Thomas Vauvert F. Hviid

**Affiliations:** ^1^Department of Clinical Biochemistry, Centre for Immune Regulation and Reproductive Immunology (CIRRI), Copenhagen University Hospital (Roskilde), University of Copenhagen, Roskilde, Denmark

**Keywords:** HLA class Ib, HLA-E, HLA-F, HLA-G, preeclampsia, immune cells

## Abstract

Despite decades of research, the highly prevalent pregnancy complication preeclampsia, “the disease of theories,” has remained an enigma. Indeed, the etiology of preeclampsia is largely unknown. A compiling amount of studies indicates that the pathological basis involves a complex array of genetic predisposition and immunological maladaptation, and that a contribution from the mother, the father, and the fetus is likely to be important. The Human Leukocyte Antigen (HLA)-G is an increasing focus of research in relation to preeclampsia. The HLA-G molecule is primarily expressed by the extravillous trophoblast cells lining the placenta together with the two other HLA class Ib molecules, HLA-E and HLA-F. Soluble isoforms of HLA-G have been detected in the early endometrium, the matured cumulus–oocyte complex, maternal blood of pregnant women, in umbilical cord blood, and lately, in seminal plasma. HLA-G is believed to be involved in modulating immune responses in the context of vascular remodeling during pregnancy as well as in dampening potential harmful immune attacks raised against the semi-allogeneic fetus. In addition, HLA-G genetic variants are associated with both membrane-bound and soluble forms of HLA-G, and, in some studies, with preeclampsia. In this review, a genetic contribution from the mother, the father, and the fetus, together with the presence and function of various immune cells of relevance in pregnancy are reviewed in relation to HLA-G and preeclampsia.

## Introduction

Preeclampsia is believed to develop in two stages: a pre-clinical stage without symptoms typically characterized by poor placentation, and a clinical stage occurring some point after 20 weeks of gestation with symptoms of increased blood pressure accompanied by proteinuria. Subclinical changes include placental oxidative stress and endothelial activation.

A unique subset of cytokine-producing decidual NK (dNK) cells is identified in the placenta during pregnancy. In contrast to the conventional NK cells of the periphery (pNK), which make up 5% of the peripheral leukocyte population, dNK cells are enriched in the placental compartment constituting up to 75% of the placental leukocyte population (1, 2). dNK cells are known to produce angiogenetic factors, and the poor trophoblastic vascular remodeling of the spiral arteries in preeclampsia has been attributed a decrease in dNK cell numbers and/or abrogated functions. Moreover, T and NK cells of the periphery are known to be activated in preeclampsia (3).

The human Major Histocompatibility Complex (MHC) is a large gene family located on chromosome 6. It includes the classical Human Leukocyte Antigen (HLA) class Ia and II genes (HLA-A, -B, -C, -DR, -DQ, and -DP). These genes and molecules are well known for their importance in antigen-peptide presentation and in organ transplantation, and for their association with a range of diseases, especially autoimmune diseases (4, 5). However, the MHC region also includes the so-called non-classical HLA class Ib genes: HLA-E, -F, and -G (6–9). The role of these genes and molecules in pregnancy and in preeclampsia is a main focus of this review.

**Figure 1:**
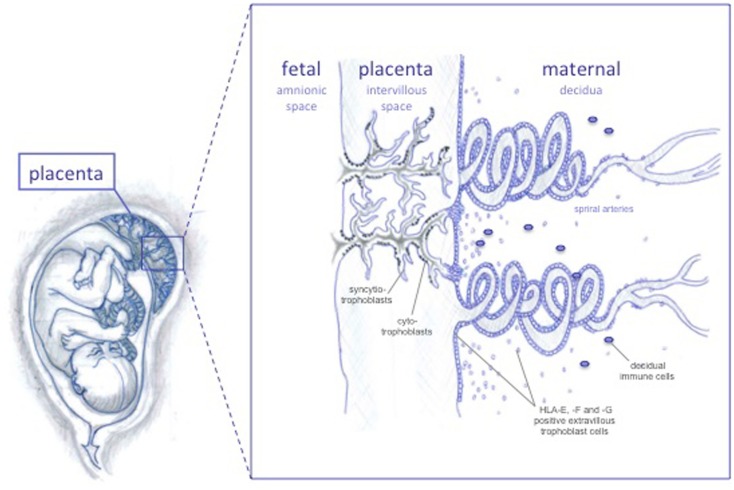
**The feto-maternal interface**. The extravillous trophoblast cells invades the maternal decidua and the spiral arteries, possibly remodeling these in order to increase blood flow to the fetus as pregnancy progresses. HLA-G and HLA-E protect invading trophoblast cells from lysis by NK cells throughout pregnancy, while HLA-F is expressed on the surface of extravillous trophoblast cells at later stages.

There are two anatomical contact-points between the maternal immune cells and the fetus: the systemic immune response between maternal circulating immune cells and the syncytiotrophoblasts, and the local immune response between decidual immune cells and the extravillous trophoblast cells (Figure 1) (10). The syncytiotrophoblast cells are devoid of HLA I molecules (11), and it is unlikely that T cell responses are directed against these. Protection from NK lysis is provided by the non-classical HLA class Ib molecules, HLA-E and HLA-G, which are highly expressed in extravillous trophoblast cells lining the placenta, and possibly also expressed by syncytiotrophoblast cells (12, 13). However, in addition to expressing the HLA class Ib molecules, extravillous trophoblast cells express low amounts of the polymorphic HLA-C, which could serve as a source of allorecognition by maternal immune cells.

## HLA Class Ib in Pregnancy

Human trophoblast cells express one HLA class Ia molecule (HLA-C) and all HLA class Ib molecules (HLA-E, -F, and -G) (6, 12, 14). Considering the unique co-expression of HLA-E, -F, and -G in the placenta and their mutual involvement in immune modulation, a combined effect or interaction of all three class Ib molecules would not seem far stretched to hypothesize (12). HLA-G has been intensively studied, HLA-E moderately studied, while little is known about HLA-F. Nonetheless, some studies on the expression and function exist, and can be related to their possible role in pregnancy.

Human leukocyte antigen-G is strongly expressed throughout pregnancy, both in the cytoplasm of extravillous trophoblast cells and on the cell surface (15, 16). HLA-F is weakly expressed in the extravillous trophoblast during the first trimester of pregnancy (16). From second trimester and on, the expression increases continuously and HLA-F translocates to the cell surface. HLA-E expression is similar to HLA-F, but HLA-E is additionally found on the cell surface in the first trimester. The increase in HLA-E and HLA-F expression coincides with fetal growth (16), and implies a role, at least for HLA-F, in this context.

**Figure 2:**
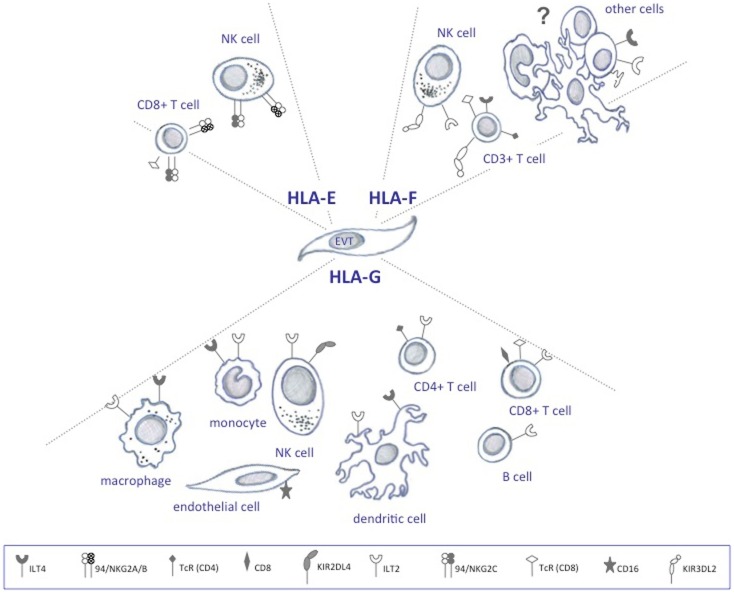
**HLA class Ib and cognate receptors expressed on decidual immune cells**.

Unlike classical HLA Ia molecules, the primary role of HLA-G is not antigen presentation, but rather immune regulation through the receptors ILT2, ILT4, and KIR2DL4 (Figure 2) (17–19). HLA-E mRNA has been detected in all cells and tissues examined and its function is likely to extend that of pregnancy (20). In contrast to HLA-G, HLA-E has been demonstrated to present antigens to a restricted subset of T cells (21), and in addition, to act as a ligand for the NK-specific CD94/NKG2 lectin receptors that regulate the activity of these cells (Figure 2) (22, 23). In the placenta, ligands for HLA-E are restricted to leader peptides from HLA-G and HLA-C, partly because of its hydrophobic properties, which limit the selection of peptides it can bind (24).

The functional role of HLA-F is the least defined. HLA-F is not believed to act in antigen presentation as it is expressed on the surface of proliferating viral-transformed lymphoid and monocyte cells without bound peptide (25, 26), and sometimes found associated with other HLA class I molecules also devoid of peptide as open conformers (27). The functional relevance of open HLA class I conformers is unclear, but it is possibly related to their unusual ability to cis-associate with themselves and other receptors (28). At least some studies indicate that these forms enable them to act as regulators of ligand–receptor interactions (28). Interestingly, similarly to HLA-G, HLA-F tetramers are able to bind ILT2 and ILT4 (Figure 2) (29).

## HLA-G Gene and HLA-G mRNA and Protein Isoforms

Eighteen HLA-G alleles have been described at the protein level according to the WHO Nomenclature Committee for Factors of the HLA System and the International Immunogenetics Information System (IMGT)/HLA Database. HLA-G exhibits low nucleotide variability in the coding regions. Most HLA-G polymorphisms do not alter the amino acid sequence, and are not expected to affect secondary structures of the heavy chains. HLA-G is alternatively spliced to produce seven mRNA isoforms, four of which encode membrane-bound protein isoforms (HLA-G1, -G2, -G3, and -G4) and three that encode soluble protein isoforms (HLA-G5, -G6, and -G7) (30–34). HLA-G1 represents the full-length isoform. HLA-G2 results from the removal of exon 3. HLA-G3 results from the removal of exon 3 and 4, and HLA-G4 from out-splicing of exon 4. HLA-G5 and -6 are soluble isoforms due to inclusion of intron 4 in the mature mRNA, which leads to secreted proteins with additional 21 amino acids encoded by the intron 4 sequence (31). HLA-G7 includes exon 2 and part of intron 2, and is predicted to encode a small soluble isoform, however, more studies are needed to demonstrate the presence of this isoform *in vivo* (34). With relevance for pregnancy, HLA-G4 and -7 mRNAs are not abundant in placentas (35).

Human leukocyte antigen-F and HLA-E have, like their counterpart, a low degree of polymorphism (36, 37). Compared to HLA-G and -E, HLA-F is distinguished in the literature by lacking exon 7, which produces a protein with a shortened cytoplasmic domain. However, HLA-G also lacks exon 7, and a newer interpretation of the intron and exon nomenclature of the HLA-G gene is currently receiving attention.

## Source of HLA Class Ib and Cellular Localization in the Placenta

Soluble HLA-G in the maternal circulation is predominantly produced and shed from trophoblast cells during pregnancy, but a quantity of sHLA-G is possibly produced by regulatory T cells and antigen-presenting cells like monocytes and dendritic cells (DCs) derived hereof (14, 38, 39). In non-pregnant individuals, sHLA-G likely reflects expression from monocytes (40, 41). Other tissues or biological fluids where HLA-G has been detected include the matured cumulus–oocyte complex, thymus, follicular fluid, and seminal plasma; furthermore at immune privileged sites, HLA-G expression has been confirmed to the eye, brain, testis, the epididymis, and the prostate gland (42–46). Also, HLA-G is secreted by erythroblasts (47), which is interesting as increased fetal erythroblastosis is detected in women who subsequently develop preeclampsia (48).

Human leukocyte antigen-E mRNA expression has been detected in virtually all cells and tissues examined and is expressed on the surface of a wide variety of cells (20).

Cellular localization of HLA-F is verified in the placenta (12), the tonsils, spleen, bladder, skin, thymus tissue, and liver cell lines (25, 49). While surface expression is absent in most tissues (25), surface expression has been demonstrated on trophoblast cells during later stages of gestation (12).

Human leukocyte antigen-G mRNA transcripts have been detected in first trimester and at term in extravillous (12, 15) and in syncytiotrophoblast cells (12), in the latter case, only mRNA transcript encoding the non-membrane forms have been confirmed (12). Because HLA-G is highly homologous to other HLA class I molecules, specific antibodies have been difficult to develop (50), and the protein expression of soluble HLA-G isoforms by syncytiotrophoblast cells cannot be ruled out, as sporadic patches with HLA-E expression have been detected in this trophoblast cell fraction (12, 13), which probably requires availability of leader peptides from HLA-G. Thus, the exact HLA-G expression profile in the syncytiotrophoblast cells is still a controversial issue.

In the placental choriocarcinoma cell line JEG-3, a physical co-localization of HLA class Ib was evidenced, showing HLA-E, -G, and -C on the cell surface, while HLA-F expression was confined to the cytoplasm (51). Also, using cell bio-imaging, a recent study revealed that HLA-G and HLA-E are co-localized in preimplanted embryos (52), indicating a prerequisite for co-expression of HLA class Ib molecules, which also could apply in the uterine compartment.

## HLA-G Conformational Variants and High Molecular Weight Complexes

A recombinant HLA-G protein consisting of the α1 and α2 domains was synthesized to mimic the extracellular part of HLA-G2 and HLA-G6 in one study (53). It showed that this HLA-G protein bound ILT4, but not ILT2, and was the first to report a binding of a HLA-G receptor with truncated HLA-G isoforms. In continuation of these findings, it was demonstrated that the same structure is able to induce tolerance and prolong the endurance of skin allografts in B6-mice and in an ILT4-transgenic mouse model (53).

In one study, HLA-G5 was hypothesized to indirectly regulate trophoblast invasion by binding to decidual leukocytes and inducing cytokine production, and as a consequence positively affect placentation (54). More specifically, recombinant HLA-G5 (rHLA-G5) was demonstrated to stimulate trophoblast invasion upon binding to KIR2DL4 and ILT2, which led to activation of the ERK pathway via phosphorylation of ERKs (54). Accordingly, trophoblast invasion was reversed with blocking antibodies for ILT2 and KIR2DL4 (54). Since insufficient trophoblast invasion is a characteristic of preeclampsia, it would be interesting if further studies of the effects of HLA-G5 on placentation were performed.

Recently, high molecular weight HLA-G complexes circulating in exosomes were identified (55). Trophoblast-derived exosomes are endocytic nanoparticles (<100 nm) shed from the placenta into the circulation, where they may stimulate or inhibit peripheral immune cells, while simultaneously expose paternal antigens systemically (56). Interestingly, the HLA-G complexes reported in exosomes were heterogeneous in nature, some proteins corresponding to ubiquitinated HLA-G, while other structures exhibited unclassified protein modifications (55). HLA-G protein alterations may affect quantification in biological fluids. Indeed, soluble HLA-G is readily detected in EDTA-stabilized blood plasma using a specific ELISA and the MEM-G/9 antibody, while the detection level is decreased in heparin-stabilized blood plasma and in serum samples (own unpublished observations). This may have important implications for detection of sHLA-G and possibly sHLA-E in the circulation of preeclamptic women, specifically when assessing their potential as biomarkers, and could explain some of the discrepancies in soluble levels previously described between studies.

Human leukocyte antigen-G exists in different forms, commonly as a monomer associated with or without the β_2_m-subunits or as hetero- or homodimers, but unique trimeric and oligomeric forms have also been acknowledged (57–59). The physiological significance of different forms remains unclear. Recent reports have demonstrated that β_2_m-associated HLA-G monomers comprise the majority of all HLA-G forms expressed by trophoblast cells (53), but a significant fraction exists in the form of HLA-G homodimers by forming an intermolecular disulfide bridge between two cysteine residues of the α1 domains of two HLA-G molecules (60). So far, the homodimer form has shown to be the most active arrangement with a higher affinity for ILT2 and ILT4 compared with the monomer (18). Furthermore, the homodimer enhances the ILT2-mediated signaling at the cellular level (18). Interestingly, in trophoblast cell lines, cell bio-imaging showed that app. 40% of HLA-E and HLA-G are co-localized in the form of tetramers or higher-order homodimer clusters (51, 52) and that HLA-E and -G form heterotypic associations with HLA-C (51), indicating a physical association on the cell surface in higher-order complexes. If these findings reflect a co-dependency of HLA-E and -G surface expression and co-localization, then a possibly reduced level of HLA-G in preeclampsia – in addition to reducing availability of leader peptide necessary for stable HLA-E surface expression – could also affect the functionality of HLA-E by other means.

Similar to HLA-G, HLA-F exists with and without association with β_2_m, and can form homodimers as well as associate with other HLA class I (25, 26). The possibility that HLA-F heavy chains have hidden functions that are determined by the amino acid sequence of the α domains is plausible (28) and should be investigated in relation to receptor–ligand interactions in pregnancy and preeclampsia.

## HLA-G in Pregnancy and Preeclampsia

Elevated levels of sHLA-G have been observed in the maternal circulation during pregnancy (61–64). An association between HLA-G and preeclampsia is supported by several findings. First, a direct association between reduced HLA-G expression in term placentas and preeclampsia has been demonstrated with *in situ* hybridization, immunohistochemistry on frozen sections, and with a ribonuclease protection assay (65–67). Second, circulating sHLA-G levels are decreased in preeclampsia, and in some cases this is observed as an early event in pregnancy in women who subsequently develop preeclampsia compared with women with uncomplicated pregnancy (62, 64, 68–70). Third, HLA-G polymorphisms have been associated with sHLA-G levels in peripheral blood from blood donors and with HLA-G protein expression in the placenta during pregnancy (71, 72), and fourth, HLA-G polymorphisms, some of which are associated with circulating levels, are further associated with increased risk of preeclampsia in some studies (73–76) but not in all (77–80). While the beneficial role of HLA-G is recognized in relation to pregnancy, a precise relationship between HLA-G and preeclampsia needs further appraisal.

## Functional Significance of HLA-G Isoforms in Relation to Pregnancy and Preeclampsia

To emphasize the function of HLA-G in relation to pregnancy and preeclampsia, several questions need to be addressed. First, which cells express cognate receptors and what is their function, second, does HLA-G exhibit isoform-specific functions, and third, what molecular structures can HLA-G form, and could it have functional relevance?

ILT2 and ILT4 are the major receptors for HLA-G. Since ILT2 and ILT4 are expressed by leukocytes – the former by most leukocytes, and the latter primarily by monocytes, macrophages, and DCs – most attention has been drawn to the interaction between HLA-G and immune cells (81). However, novel functions of HLA-G have been suggested, possibly in the context of vascular events during placentation. Indeed, both ILT2 and ILT4 have been identified in the mesenchyme of term placentas, but with different localization. ILT2 was abundant in stromal cells, while ILT4 was prominent in perivascular smooth muscles. Interestingly, trophoblast cells express neither receptor (82). This is consistent with recent findings showing that HLA-G5 dimers engage with ILT4 in airway smooth muscle (83). Although ILT2 may be the major binding protein for leukocytes, ILT4 has been suggested as the main receptor for HLA-G. Additionally supporting an alternative role of HLA-G is the observation that CD160, an sHLA-G1 receptor found on endothelial cells but not reported on trophoblast cells, inhibits angiogenesis by an apoptotic pathway (84).

Arguments for existence of HLA-G-isoform-specific functions include the observation that HLA-G2 and -G6 isoforms are expressed exclusively in the extravillous trophoblast cells distal to the villous, while HLA-G5 is ubiquitously expressed in syncytiotrophoblast cells (85, 86) and maternal blood (62). The major isoform-specific distinction supported by experimental studies is based on a functional concentration-dependency, which implicates HLA-G5 as a potentially more effective stimulator according to some studies (59, 87). HLA-G5 expression in the placenta seems to be sparse, at least at the mRNA level (50, 88, 89). Moreover, an isoform-specific role for HLA-G5 in relation to pregnancy was indicated in a recent study where HLA-G5 – while low or completely absent in maternal blood at term in normal pregnancies – was significantly increased in preeclampsia (62).

On the other hand, an argument for similar functions between different HLA-G isoforms is given by studies that describe women who are homozygous for the HLA-G*01:05N null allele (597DeltaC) and thereby lack expression of HLA-G1 and -G5. However, they have demonstrable HLA-G levels in the placenta and produce viable offspring, which is consistent with the idea that other isoforms – or other HLA class Ib molecules – provide functional compensation (90).

Most studies correlating circulating sHLA-G levels with preeclampsia have focused on the HLA-G1 and -G5 isoforms, which are nearly identical. Soluble HLA-G1 is derived from the full-length membrane-bound isoform containing a transmembrane cytoplasmic region, which may be cleaved by metalloproteases and shed from the cell surface (91, 92).

The soluble isoform HLA-G5 is generated due to a stop codon in intron 4 that prevents translation of the transmembrane cytoplasmic domain. Due to technical challenges, HLA-G5 has long been difficult to identify with specific monoclonal antibodies, but this issue seems lately to have been overcome (62). One argument for focusing on HLA-G1 is that it represents the most abundant isoform in the placenta. However, a functional distinction among HLA-G isoforms is plausible.

Human leukocyte antigen-G1 is by far the most abundant HLA-G mRNA isoform, both in preeclamptic placental biopsies and control placental biopsies, followed by G3, G5, G2, and G6 (35, 88). HLA-G4 and -G7 mRNA transcripts are not abundant in placentas (35). An *in vitro* functional study showed that the truncated isoforms G2, G3, and G4 are expressed on the surface of transfected cells and protect against NK and T cell-mediated cytotoxicity (93), and more recently a transfection study showed that HLA-G1 and HLA-G3 differentially increased HLA-E surface expression (94), indicating that the less abundant HLA-G isoforms are able to functionally compensate for HLA-G1 but with different effectiveness. However, low transcript abundance and/or protein expression in the placenta has prompted researchers to assume that these transcripts are less relevant, and *in vivo* relevance is typically only supported for G1 and G5. Interestingly, a study found that the HLA-G mRNA profile in term placental biopsies is shifted toward a higher frequency of HLA-G5 in preeclampsia (35), which is supported by higher HLA-G5 protein levels in maternal blood in preeclampsia compared to controls according to another, independent study (62).

## HLA-G Polymorphisms Linked to Preeclampsia

A 14 bp insertion/deletion (ins/del) HLA-G polymorphism in the 3′ untranslated region (3′UTR) first described by Harrison et al. (95), is the best studied HLA-G polymorphism and has shown to influence HLA-G mRNA transcript size and stability (31, 88, 96–98).

Preeclampsia is a pregnancy condition unique to humans (99). The HLA-G 14 bp deletion allele is also unique to humans (100), and interestingly, this allele is more prevalent than the insertion allele (101, 102), raising the question whether the 14 bp deletion variant evolved evolutionary as a compensatory mechanism to counter pathological conditions only seen in humans. It is an intriguing thought that this theory could apply to preeclampsia.

Several studies have been undertaken in effort to clarify, whether the fetal HLA-G 14 bp ins/del genotype predisposes to preeclampsia in the mother (Table 1). One study found an association between the 14 bp insertion allele in offspring from primiparous preeclamptic women and controls (76, 103), which was supported by another study that further demonstrated a reduced level of the G3 isoform in placentas homozygous for the insertion in mild preeclampsia (73). Conversely, other studies found no association in offspring cases of preeclampsia, but noteworthy, included women with different degrees of preeclampsia (78, 104, 105). The discrepant results from different studies leave the influence of the fetal 14 bp ins/del genotype on the risk of developing preeclampsia controversial. However, published studies are characterized by small sample sizes, and larger scale studies are necessary. Furthermore, assessing combined mother–child HLA-G genotypes may be a better approach. The above mentioned case-control study of 155 family triads of mother, father, and offspring performed by Hylenius et al. showed an association of homozygosity for the 14 bp ins allele in offspring from primiparous women with severe preeclampsia (103), also supported by others (104, 106). Furthermore, the results suggested that a 14 bp ins/del contribution from the father influenced the risk of developing preeclampsia (103).

**Table 1:** **Summary of previous studies investigating possible associations between HLA-G polymorphisms/alleles and preeclampsia**.

Study	Study size (case/control)	Parity subjects (case/control)	Subject	Association with preeclampsia
**14 bp ins/del polymorphism**
Bermingham et al. (105)	68/74	Primiparous: all	Parents and offspring	No
O’Brien et al. (73)	7/11	ND	Offspring	Yes
Hylenius et al. (103)	57/98	Primiparous: 40/70 Multiparous: 17/28	Parents and offspring	Yes. Association in offspring and in mother/offspring pairs. Association with paternal inheritance (only significant in primiparous cases)
Vianna et al. (77)	157/162	ND	Mothers	No. A trend showing higher allele frequency of 14 bp del in mothers with preeclampsia
Moreau et al. (74)	36/60	ND	Offspring	Yes
Iversen et al. (78)	31/43	ND	Mothers and offspring	No
Zhang et al. (106)	120/158; 82/87; 67/75	ND	Mothers and offspring; parents; fathers and offspring	Yes. Association in offspring, in mother/offspring pairs and father/offspring pairs
**+3187 polymphism**
Yie et al. (75)	29/15	Nulliparous	Offspring	Yes
G*01:04:xx
Carreiras et al. (207)	104/29	ND	Mothers and offspring	Partly, when the allele was maternally inherited
Hylenius et al. (103)	57/98	Primiparous: 40/70	Parents and offspring	No
		Multiparous: 17/28	
G*01:05N
Aldrich et al. (79)	57/36	ND	Offspring	No
Hylenius et al. (103)	57/98	Primiparous: 40/70	Parents and offspring	No
		Multiparous: 17/28	
Loisel et al. (111)	58/314	ND	Mothers	Yes
G*01:06
Moreau et al. (74)	36/60	ND	Offspring	Yes
Tan et al. (104)	83/240	Primigravidas: 20/92	Mothers and offspring	Yes. Also when paternally inherited (multiparous women)
		Multigravidas: 63/148	

*ND, not determined/not described*.

A puzzling thing about the 14 bp ins/del polymorphism is the controversy about the abundance, and possibly, stability of the two alleles. In fact, as stated earlier, the mRNA deletion transcript has been shown to be more abundant than the mRNA insertion transcript. This fits well with studies showing higher sHLA-G levels when homozygous for the deletion, and importantly, with studies that support an association between the insertion allele, reduced HLA-G levels and preeclampsia (72, 88). A mechanism that might be compensatory to the lower HLA-G protein expression associated with the insertion allele exists: the presence of an alternative splice transcript produced from, and secondary to, the 14 bp insertion mRNA transcript. An *in vitro* study inducing a transcriptional stop with Actinomycin D treatment in JEG-3 and M8 cell lines, showed that the alternate transcript, characterized by removal of 92 bases from the insertion transcript, is more stable than the 14 bp insertion transcript (96). However, the −92 bp variant does not represent the majority of transcripts (88, 96), and its physiological relevance *in vivo* remains to be investigated. Complicating the matter of linking differential HLA-G protein expression to either the insertion or deletion mRNA transcripts, a recent study using a K562 cell line transfected with the insertion and deletion sequences separately, reported that membrane-bound HLA-G was higher in insertion transfectants, while sHLA-G was lower (98). Although these findings need verification, the study by Svendsen et al. indicates that the 14 bp ins/del genotype could have an impact on the soluble/membrane-bound HLA-G ratio, and could help clarify some of the conflicting results from preeclampsia studies. As a highly debatable explanation to the findings by Svendsen et al., ins/del HLA-G mRNA transcripts could have different structural features of the untranslated regions and coding sequences – a major and overlooked part in the control of mRNA translation. Relaxed secondary structures in UTRs are common for many mRNAs and characterize transcripts that are translated at a high rate (107). Conversely, more stable mRNA secondary structures containing e.g., hairpin loops, although exhibiting low turnover of mRNA, may be translated at a slower rate (107). The secondary structures of the 14 bp ins/del mRNA transcripts have not been elucidated, but potential differences could explain why the insertion allele, albeit less abundant, is associated with high membrane-bound HLA-G. It does not, however, explain the lower sHLA-G levels associated with the insertion allele, which could be related to differences in the dynamics of HLA-G translation and post-translational mechanisms, e.g., shedding of HLA-G1 from the cell surface.

Several HLA-G SNPs are shown to be in strong linkage disequilibrium with the 14 bp ins/del polymorphism. These include a −725 SNP located in the promoter region previously shown to affect the transcriptional rates of HLA-G (108), and an array of SNPs in the 3′UTR downstream from the 14 bp ins/del that may act as microRNA sites and influence mRNA size and stability (109, 110). These include SNPs at +3142, +3187, and +3196 (109). Yie et al. reported that the +3187 SNP was associated with differences in mRNA stability, and that homozygous offspring were strongly correlated with severe preeclampsia (75). An association between HLA-G haplotypes and preeclampsia has been reported in some studies (76) but not in all (111). In the study by Larsen et al., a fetal HLA-G 3′UTR haplotype consisting of the 14 bp insertion sequence, a C at the +3010 SNP, a G at the +3142 SNP, an A at the +3187 SNP, and a G at the +3196 SNP was associated with the risk of developing severe preeclampsia in primipara (76). Interestingly, another fetal HLA-G 3′UTR haplotype with the 14 bp deletion, a G at the +3010 SNP, a C at the +3142 SNP, an A at the +3187 SNP, and a C at the +3196 SNP was much more frequent in the control group of primipara with no preeclampsia compared to the primipara group with severe preeclampsia (26.4% vs. 6.3%).

An HLA-G allele containing the 14 bp insertion, G*01:06, has been linked to preeclampsia in different studies [(74, 103, 104)]. The polymorphic 1 bp deletion of a cytosine residue at codon 130 which results in null allele (G*01:05N) described earlier, is associated with increased risk of preeclampsia in one study (111), and a reduced HLA-G level in maternal serum from normotensive African-American controls was observed in women bearing the null allele (111). However, this was not confirmed in another study (79). The 1597ΔC null mutation is rare in Europeans but more common in other global populations (79, 102, 112, 113), which emphasizes that ethnic difference or demographic factors should be considered in future study set-up, or when interpreting meta-studies on the association of HLA-G polymorphisms with preeclampsia.

Taken together, whether HLA-G genotypes and expression patterns might have a significant influence on the development of preeclampsia remains controversial. Further studies investigating an array of polymorphisms associated with preeclampsia in a larger scale are warranted, especially ones that set to investigate the mRNA and cell surface protein expressions simultaneously.

## HLA-E Allelic Polymorphisms

Two non-synonymous HLA-E alleles, E*01:01:xx:xx and E*01:03:xx:xx, have been identified (36, 114). They are distinguished by having either an arginine or a glycine at position 107 of the protein, and are so far the only HLA-E allelic variants to affect intracellular trafficking and surface expression (115). The frequency of these alleles is nearly equal in different populations, which indicates a balancing selection implying that a functional difference exists between the two alleles (116). One study showed that, although no difference was found between proteins in steady-state, the E*01:03:xx:xx allele exhibited higher surface expression than the E*01:01:xx:xx allele (117). In addition, the E*01:01:xx:xx and E*01:03:xx:xx alleles differ in their peptide binding affinities, E*01:03:xx:xx exhibiting a 10- to 100-fold higher affinity than E*01:01:xx:xx. A differential expression could have consequences for the inhibitory effect of HLA-E on NK cells and T cells. Indeed, the surface levels of HLA-E have been shown to affect inhibitory activity *in vitro* (22), and HLA-E polymorphisms have been associated with nasopharyngeal carcinoma (118), and recurrent spontaneous abortions (119). If HLA-E expression is hypothesized to be important in the context of pregnancy, an association of preeclampsia with HLA-E polymorphisms seems relevant to investigate. While no such study exists, one study showed that sera from early-onset, severe preeclamptic women could induce HLA-E surface expression in an EA.hy296 endothelial cell line *in vitro* (120). This upregulation was countered by addition of recombinant interferon (IFN)-γ. Soluble HLA-E was detectable in sera, but no difference was found between preeclamptic women and controls (120), indicating HLA-E surface expression on endothelial cells as a symptom of endothelial activation in preeclampsia, possibly mediated by other factors.

## Paternal Contribution to Preeclampsia

Preeclampsia is mostly considered a disease with maternal and fetal involvement, but there are some indications of paternal contributions as well. For example, preeclampsia is associated with an increased partner-specific CTL response in a mixed lymphocyte reaction (MLR), a finding that was not observed, when the MLR was performed with an unrelated partner, who fathered two previous uncomplicated pregnancies (121). This study indicates a maternal response directed against specific paternal antigens. In addition, the fetus is a natural allograft and the mother could carry killer immunoglobulin-like (KIR) allelic gene variants that mismatch with paternal HLA-C expressed on trophoblast cells. KIR receptors constitutes a highly polymorphic family of HLA class I receptors expressed on NK cells that is able to engage a cytotoxic NK cell response upon binding to HLA-C in the placenta. One study found that the combination of maternal KIR-AA and fetal HLA-C2, but not fetal HLA-C1, lead to increased risk of preeclampsia (122), but more studies are needed to confirm this.

A paternal contribution of the G*01:06 allele increases the risk of preeclampsia in multigravidae, at least according to one study (104). In the case-control study using family triads by Hylenius et al., an importance of paternal transmission of the 14 bp ins HLA-G allele to the offspring in the preeclampsia triads was observed, which supports the findings by Tan et al. (103). Another triad-study found that father/offspring pairs homozygous for the 14 bp del were significantly less frequent in early- compared to late-onset preeclampsia (106).

## Immune Cells in Pregnancy and Preeclampsia

**Figure 3:**
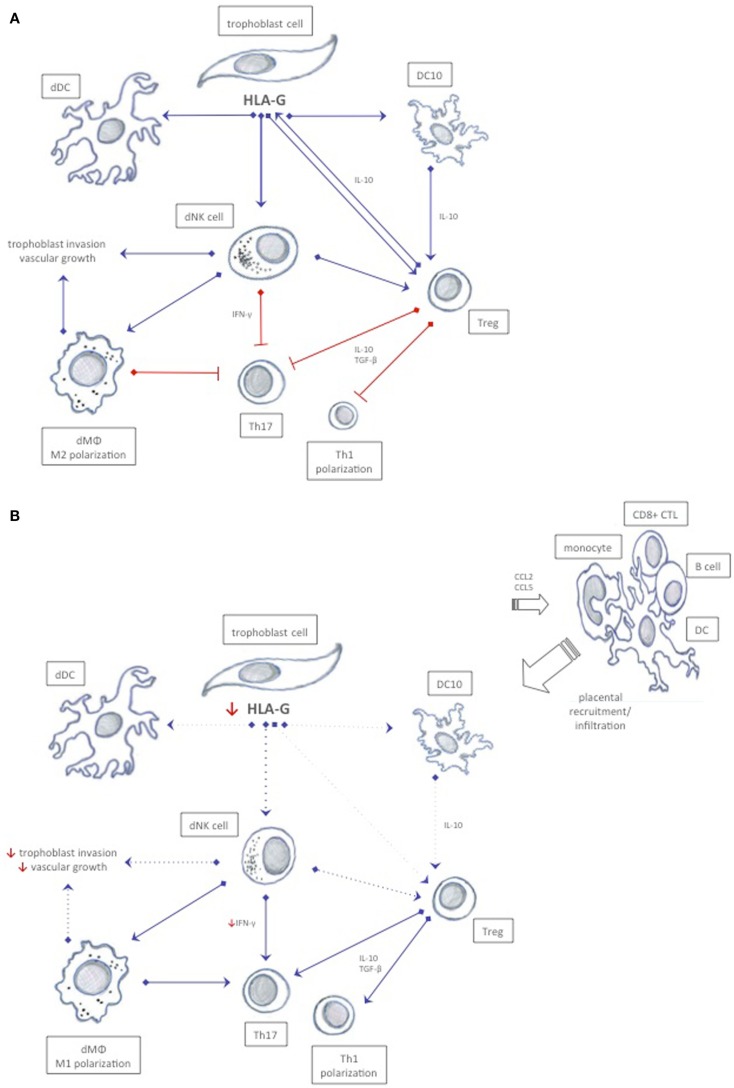
**Possible immune interactions between HLA-G and decidual immune cells in normal pregnancy and in preeclampsia**. **(A)** In normal pregnancy, HLA-G expression is believed to ensure a tolerogenic uterine environment by inhibiting cytotoxicity, inducing release of anti-inflammatory cytokines, and by promoting proliferation of tolerogenic decidual immune cells that mutually stimulate each other to sustain tolerance. (**B**) In preeclampsia, a possible reduced soluble and membrane-bound HLA-G expression in trophoblast cells may affect immune cells expressing cognate receptors, and thus enhance immunity rather than tolerance. Increased CCL2 and CCL5 chemokines and inflammatory cytokines may recruit activated immune cells from the periphery further abrogating the tolerogenic milieu. Dotted lines represent reduced stimuli.

Initially, data from epidemiologic studies suggested that inappropriate activation of the immune system or immune maladaptation plays a critical role in the development of preeclampsia (123). *Ex vivo* studies have since confirmed that immune cells play a central role in the pathophysiology of preeclampsia (124). An emerging theory is that a shift in immune cell functionality in uterine subpopulations reflects a maladapted maternal immune system, or a loss of tolerance mechanisms, which precedes the progress of placental oxidative stress and ischemia observed in preeclampsia (Figure 3) (125). Uncomplicated pregnancies are dependent on a delicate interplay between regulatory T cells and dNK cells that recognize and accept paternal antigens presented by the semi-allogenic fetus while simultaneously allowing vascular remodeling and placental growth (3). Although regulatory T cells and dNK cells have been the focus of most studies, it is likely that other immune cells like monocytes, DCs, and macrophages participate in upholding fetal tolerance (Figure 3). An aberrant/activated maternal immune system is associated with pregnancy complications like recurrent spontaneous abortions and preeclampsia. The expression of HLA-G receptors on decidual immune populations like NK cells, T cells, DCs, monocytes, and macrophages implicate HLA-G in the regulation of the uterine microenvironment (126, 127). However, direct effects of HLA-G on immune cell activation, recruitment, and function in the context of preeclampsia remain to be elucidated.

## NK Cells in Pregnancy and Preeclampsia

The early decidua is characterized by a unique population of dNK cells that constitute 50–90% of all leukocytes present in the uterine compartment in first trimester (1, 128). Compared to conventional pNK cells circulating the periphery, dNK cells exhibit a different repertoire of cytokines and receptors reflecting a more tissue-specific function (128, 129). dNK cells secrete vascular endothelial growth factor (VEGF), placental growth factor (PLGF), interleukin-8 (IL-8), and IFN-inducible protein-10 (IP-10) (129). In an *in vitro* migratory assay, dNK cell migration was correlated to the amount of the chemokines IL-8 and IP-10, when co-cultured with trophoblast cells (129), indicating a specific recruitment possibly mediated by the cognate CXCR1 and CXR3 chemokine receptors expressed on trophoblast cells. An aberrant production of cytokines and chemokines could have a great impact on the depth of trophoblast infiltration/invasion as seen in cases of preeclampsia.

In preeclampsia, pNK cells have en altered NKG2A and -C receptor expression (130), while dNK cells isolated from decidua at term show a higher expression of NKG2-associated receptor CD94 (131). HLA-G interacts with three inhibitory receptors, ILT2, ILT4, and KIR2DL4, as discussed earlier (132, 133). KIR2DL4 is not expressed on the surface of NK cells in steady-state, but surface expression can be induced after *in vitro* culture, and the expression and function is determined by genotype (134). KIR2DL4 seems not to be associated with preeclampsia. However, the presence of a fetal G*01:06 allele in combination with the maternal KIR2DL4*006 allele has been reported to be significantly associated with preeclampsia risk in multigravida pregnancies, suggesting a gene–gene interaction (135).

A recent study showed that a decidual population of CD56^high^CD27^+^ dNK cells accumulates in the first trimester of pregnancy and dampens the effects of inflammatory Th17 cells via IFN-γ secretion (136). In an Nfil3^−/−^ mouse model of pregnancy where the mice lack NK cells entirely, and in an NK cell-depleted pregnant mouse group, they both demonstrated a significantly higher percentage of Th17 cells (136). In humans, the CD56^high^CD27^+^ dNK cells and their supernatants inhibited the expansion of Th17 cells – an effect reversed by addition of neutralizing anti-INF-γ (136).

There is still some controversy about NK numbers in preeclampsia. In peripheral blood, the prevalence of NK cells differ between preeclamptic cases and controls in some studies (137) but not in all (138). However, it is more likely that a difference should be found in the uterine environment within the dNK population. HLA-G has been shown to inhibit NK lysis in HLA-G transfected cell lines in a concentration-dependent manner (91, 139, 140), and the physiological relevance of this effect was demonstrated by a study showing that *ex vivo* NK cell functional responses to HLA-G differ between peripheral blood and decidua, where dNK cells were refractory to stimulation compared to pNK cells (141), further supporting the important role of HLA-G in sustaining pregnancy and its influence on dNK cells.

## T Cells in Pregnancy and Preeclampsia

CD4^+^ T cells, or T helper (Th) cells, can be subgrouped on the basis of their cytokine profile into Th1 and Th2 T cells. According to an early theory, successful pregnancy is biased toward a Th2 humoral response characterized by release of immunoregulatory cytokines such as IL-10 and TGF-β (142). Cytokines and other soluble factors like progesterone and indoleamine 2,3-dioxygenase (IDO) have been proposed to act on the Th1/Th2 balance, and a shift toward a Th1 response has been hypothesized to occur in preeclampsia (143). Furthermore, when cell lines are transfected with membrane-bound HLA-G1 and co-cultured with decidual or uterine mononuclear cells, several studies have observed a decrease in TNF-α and an increase in IL-10 (144–146). So, it seems plausible that HLA-G can mediate a shift from a proinflammatory Th1 cell-mediated response toward a Th2 response inducing tolerance. However, pregnancies in Th2 knockout mice proceed without complications, indicating how a higher complexity of the cytokine network in the placenta or other mechanisms may add to fetal tolerance (147). In the slipstream of the Th1/Th2 paradigm, a new has emerged: the Th1/Th2/Th17/T regulatory cells (Tregs) paradigm (148). Th17 cells are immunoregulatory cells that play a critical role in induction of inflammation and have been linked to autoimmune diseases and tissue transplant rejection, and possibly to pregnancy complications (148, 149). The Th1/Th2 balance and the capacity of Th17 cells to produce cytokines are modulated by TGF-β and IL-10 or by cell–cell interaction with CD4^+^CD25^high^ Tregs, described later (148). Although little is known about Th17 cells, recruitment and expansion of this subset seem to be promoted by proinflammatory cytokines like IL-1β and IL-6, and the highest percentage exists in the first trimester (136, 150). Interestingly, a novel role for Th17 cells in trophoblast proliferation and invasion was recently indicated (151). In this study, Th17 cells were recruited from the periphery in early pregnancy by CCL2-secreting decidual stromal cells, and inhibited apoptosis of trophoblast cells via an IL-17-dependant mechanism (151), suggesting a vital role for Th17 cells in normal pregnancy. However, an exaggerated production of IL-17 could have unwarranted consequences. In preeclampsia, the prevalence of IL-17-producing CD4, CD8, and NK cells is elevated in peripheral blood compared with normotensive pregnant women (152), and the Th1/Th2 and Th17/Treg balance is shifted toward increased immunity determined by a Th1 response, elevated Th17 T cells and reduced Treg numbers, possibly affecting the uterine microenvironment conjointly with dNK cells (152). Furthermore, in preeclampsia, monocytes produce IL-1β and IL-6 that mediate terminal differentiation of Th17 cells possibly causing an exaggerated inflammatory response, which may consequently reduce Treg abundance and function (148).

Classical Tregs constitute a subset of T cells with suppressive properties. They are capable of inhibiting redundant immune responses in a very potent fashion, and aid in maintaining antigen-specific T cell tolerance important in pregnancy (153). In mice, the Treg population increases markedly during early gestation (154), and a similar effect is observed in pregnant women with a peak during the second trimester and a decline in numbers postpartum (155). Adoptive transfer studies in mice have demonstrated the physiological importance of CD4^+^CD25^+^ Tregs in pregnancy (156, 157). For example, when a total pool of CD4^+^ T cells is depleted of the CD4^+^CD25^+^ Treg subpopulation and transferred into pregnant mice deficient of T cells, allogeneic mice fetuses are rejected, while syngeneic fetuses remain unaffected (156). In humans, isolated CD4^+^CD25^+^ cells are able to suppress autologous CD4^+^ T cells stimulated by allogeneic DCs (155), and to inhibit IL-4 secretion against paternal but not unrelated allo-antigens *in vitro* (158).

In preeclampsia, the number of CD4^+^CD25^high^ Tregs is decreased in peripheral blood (150, 159) as well as in term placentas (160). However, not all studies confirm these findings (161). Assessing Treg numbers based on the co-expression of CD4 and CD25 solely has been questioned, and with the identification of the transcription factor forkhead box P3 (Foxp3), a more reliable marker for Tregs was found. In support of the findings associating CD4^+^CD25^high^ Treg numbers with preeclampsia, circulating levels of CD4^+^CD25^high^FoxP3^+^ Tregs are decreased in preeclamptic women (138, 150, 162). Highly relevant in the context of identifying Tregs, a study by Santner-Nanan et al. compared CD4^+^CD25^high^, CD4^+^CD127^low^CD25^+^, and CD4^+^Foxp3^+^ cells from preeclamptic women and controls, and found that the frequency of Tregs in all three “groups” was reduced in preeclamptic women (150). However, *ex vivo*-sorted Tregs had preserved their suppressive properties implying that a reduced number of Tregs rather than a lack of suppressive function occurs in preeclampsia (150). Furthermore, Santner-Nanan et al. also reported that the ratio of Tregs to Th17 was significantly increased in normal pregnancy but not in preeclampsia (150). The conversion of Tregs to Th cells has been documented in both mice and humans (163), and lately, this conversion has been suggested to occur as a part of the pathophysiology of preeclampsia (164).

Subsets of non-conventional Tregs more recently described include HLA-G-positive Tregs and tolerogenic CD4^low^ and CD8^low^ T cells. CD4^+^HLA-G^+^ Tregs lack classical Treg markers and are characterized by the constitutive expression of HLA-G (165). Functional characterization indicates that the suppressive properties of this subset rely on the immunoregulatory properties of HLA-G, which enables CD4^+^HLA-G^+^ Tregs to inhibit bystander immune activations by direct cell–cell interaction (166). In normal pregnancy, the prevalence of CD4^+^HLA-G^+^ T cells is high in decidua (167), while a recent study showed that the expansion of the HLA-G-positive T cell subset is impaired in preeclampsia (168). Furthermore, it was indicated that classical Foxp3 Tregs and CD4^+^ T cells acquire HLA-G from monocyte-derived DCs via the process of trogocytosis where membrane fragments are dispatched from the DCs and transferred to the surface membrane of leukocytes (168).

Non-conventional regulatory T cell subsets, which are distinguished by lower surface expression of CD4 and CD8, have been identified in a transplantation study (169). Interestingly, regulatory activity by these CD3^+^CD4^low^ and CD3^+^CD8^low^ T cells was induced by soluble HLA-G and/or HLA-G1-expressing DCs (169). While these subsets have not been investigated in relation to pregnancy and preeclampsia, it is reasonable to believe that their suppressive activities mediating allograft acceptance could be relevant in a pregnancy setting, and match a hypothesis where HLA-G in the placental microenvironment influences the phenotype and function of local T cells.

## B Cells in Pregnancy and Preeclampsia

In normal pregnancy, the almost complete absence of B cells in decidua suggests that no B cells are localized, recruited nor activated by fetal allo-antigens (170). Like for other leukocytes, ILT2 is also expressed on the surface of B cells (133). A recent study in mice showed that ILT2–HLA-G engagement on B cells inhibits both naïve and memory B cell function *in vivo* and *in vitro* at the level of proliferation, differentiation, and Ig secretion (171). The inhibitory effects of HLA-G were independent of the form of B cell activation, suggesting that the presence of T cells could be less important. Moreover, HLA-G mediates phenotypic and functional downregulation of CXCR4 and CXCR5 chemokine receptors on germinal center B cells (171). *In vivo* support for HLA-G as a negative B cell regulator was provided in a xenograft mouse model, which showed a significantly altered antibody secretion pattern (171).

A specific subpopulation of CD19^+^CD5^+^ B cells that secrete autoantibodies is identified in preeclampsia (172), indicating a dysfunctional immune regulation or B cell activation mediated by fetal allo-antibodies. Furthermore, a recent study on the interactions between Tregs and B cells indicated that a negative correlation between Tregs and memory B cells exists in peripheral blood of preeclamptic women (173). Although the Treg population was reduced numerically, interestingly, the suppressive effects on autologous B cell proliferation were unaffected (173).

## DCs and Monocytes in Pregnancy and Preeclampsia

In the periphery, DCs play a crucial role in linking innate and adaptive immunity by virtue of their exceptional ability to capture, process and present antigens to naïve T cells, and by mediating cross-talk with a broad range of immune cells. In the decidua, however, DCs are scarce, making up app. 1% of the decidual immune population (174). A decidual subset of tolerogenic DCs that express high levels of HLA-G was recently identified. These cells spontaneously secrete high amounts of IL-10 and are named DC-10. DC-10s can be differentiated *in vitro* from peripheral blood monocytes with proinflammatory cytokines including granulocyte macrophage-colony stimulating factor (GM-CSF), IL-4, and IL-10 (175, 176). DC-10s are able to induce immunosuppressive CD4^+^ T cells, and their potency to do so was demonstrated when a single stimulation of CD4^+^ T cells with DC-10 promoted a fraction of anergic T cells that contained up to 15% of already differentiated inducible Tregs (177, 178). In a transplantation study, engagement of ILT4 on DCs by HLA-G-tetramers resulted in maturation/activation, and prolongation of allogeneic graft survival (179). The local milieu in the placenta is likely to moderate the function and activity of local immune cells, but evidence points to a systemic effect as well. As an example, the TLR expression and cytokine profile in circulating DCs is dysregulated in preeclampsia, and they demonstrate a weaker response to TLR-stimulation compared with controls (180). In addition, a recruitment of mature and immature DCs to the decidua is observed in preeclampsia (181).

## Macrophages in Pregnancy and Preeclampsia

The majority of decidual leukocytes in the first trimester consist of NK cells and second to these are the tissue-specific macrophages, which make up 20–25% (1, 182, 183). These decidual macrophages are characterized by their immunosuppressive abilities, and two different subsets have so far been identified in the feto-maternal interface; single-positive CD14^−^CD68^+^ and double-positive CD14^+^CD68^+^ macrophages (183, 184), which, however, still need to be characterized. The abundance of decidual macrophages in the first trimester indicates vital tissue-specific functions and thus, an important role in maintenance of normal pregnancy (185). In support of this notion, they are co-localized with evading trophoblast cells and found in the vicinity of spiral arteries, where they are believed to modulate the immune response to pathogens, to mediate vascular remodeling and promote trophoblast invasion (186, 187).

Studies have shown that decidual macrophages may contribute to the development of preeclampsia, primarily by a shift in the cytokine profile leading to poor spiral artery remodeling (186, 188, 189). In addition, increased macrophage infiltration in the decidua is observed in preeclampsia (181). Upon proinflammatory stimuli, monocytes, macrophages, and DCs are recruited to the decidua by specific chemokines, especially CCL2 and CCL5 (190). In accordance with the observed increase in infiltration of macrophages in preeclampsia, CCL2 and CCL5 expression is increased in preeclamptic decidua (181). An excessive release of GM-CSF in preeclamptic placentas contributes to macrophage differentiation, further increasing the production of proinflammatory cytokines (191). TNF-α, PAI-1, and inducible nitric oxide synthase secreted by decidual macrophages inhibit trophoblast invasion and migration, and thus, spiral artery remodeling (192, 193). Macrophages regulate angiogenesis by secreting VEGF, which binds to fms-like tyrosine kinase-1 (Flt-1), both of which are dysregulated in preeclampsia (194). In addition, decidual macrophages express IL-2 and ILT4, and HLA-G may thus regulate their functional properties (82, 133). This was indicated in a study showing that upon co-culture with transfectants expressing HLA-G homodimers, cytokine production was greatly increased in CD14-positive decidual macrophages (195).

Similar to the concept of Th1/Th2 polarization in effector T cell function, macrophages are characterized according to their effector phenotype and cytokine repertoire, subgrouping them into classical activated macrophages, M1, or alternatively activated macrophages, M2 (185, 196). M1 secretes IL-12 and TNF-α upon stimuli from LPS or IFN-γ, while M2 upon stimuli with IL-4 secretes the tolerogenic cytokines IL-10 and IL-13 (196). However, the existence of a M1/M2 balance in the placenta and the possible implication of this in preeclampsia still need to be investigated. Recently, increased numbers of CD14^+^ cells were identified in preterm preeclamptic placentaes, and – supporting the importance of a M1/M2 balance – a lower CD163+/CD14+ ratio (M2), and a higher CD209+/CD14+ ratio (M1) were observed in preeclamptic placentas compared with controls (197).

## Conclusion and Perspectives

A vast amount of evidence highpoint an involvement of immune cell populations in pregnancy, and preeclampsia is indeed characterized by an aberrant immune system. While studies show that a broad continuum of immune cells are affected, or more specifically activated, to induce unwanted immunity rather than tolerance against the semi-allogeneic fetus in preeclampsia (123, 125), an important question is whether this occurrence precedes the abrogated placentation and endothelial activation and inflammation observed. Associations between cytokine production and repertoire and vascularization support this theory. Given that immune maladaptation is an early event in the etiology of preeclampsia, we speculate whether one or few immune populations are responsible for altering the local, and possibly systemic, cytokine milieu resulting in a more general change in the function and abundance of other immune cells not typically present in the uterine environment – like B cells and DCs. This would require an immune population that acts as a “linker” between the innate and adaptive immune system, and in addition, an immune population with specific receptors for HLA class Ib expressed by trophoblast cells. A simple answer would be that the dNK cells constitute this “linker” population. However, the explanation may not be that straightforward. Transplantation studies have offered new insights into tolerance mechanisms provided by other immune cells. These include tolerogenic CD4^low^ and CD8^low^ T cells, HLA-G-expressing T cells and HLA-G-expressing DCs, and in this context, the key perspective may not be abundance, since these cells are present in low numbers, but instead tolerance potency.

Immunological memory is another important aspect that needs to be addressed. According to epidemiological findings, primiparity is the strongest risk factor for preeclampsia occurring in up to 75% of cases (123, 198, 199). Furthermore, in multiparas, a change of partner increases the risk to the level of the first pregnancy, although the idea of a partner-specific effect has been challenged as merely a consequence of a long interval since the last pregnancy, which is also a risk factor of preeclampsia (123, 200). Memory T cells, which induce tolerance to paternal antigens, may explain these epidemiological findings (123, 201). In mice, an accelerated expansion of maternal CD4^+^Foxp3^+^ Tregs specific for fetal antigens support that multiparas are protected by a regulatory memory for fetal antigens (201, 202). Recent data have also revealed that exposure to seminal fluid may induce paternal-specific tolerance (203) and short cohabitation, use of condoms and insemination with donated spermatozoa are risk factors of preeclampsia (123) suggesting that absence of semen exposure could fail to induce adequate tolerance, resulting in preeclampsia. Paternal allo-antigens and soluble factors like TGF-β, prostaglandins and HLA-G are present in seminal fluid, and could well prove important for Treg expansion, differentiation, and immunological memory (42, 203, 204).

While some decidual cell populations, including Tregs and DC-10, may be licensed for tolerance induction or immune modulation even before conception, it is likely that their differentiation and proliferation is co-dependent on the HLA class Ib molecules both in the initial stages and throughout the course of pregnancy. Indeed, reviewing the idea that a “linker” is needed to affect vascularization and different immune populations simultaneously, and given that aberrant dNK function and numbers are not sufficient to account for the pathophysiology observed in preeclampsia alone, this “linker” may well be represented by HLA-G. The low expression of HLA-G in preeclampsia, and the sum of *in vivo* and *in vitro* studies showing a broad array of immune interactions/cross-talk with, and through, HLA-G and cognate receptors, supports this hypothesis. Why is it then that genetic variation in HLA-G, although nicely shown to influence the transcription and expression of HLA-G *in vitro* still lacks strong association with preeclampsia in some studies? One answer could be that we still lack knowledge of some fundamental aspects of HLA-G biology. What significance can be attributed the alternative splicing of HLA-G mRNA transcripts, and what are their isoform-specific functions? What is the significance of higher-order HLA-G- and HLA class Ib protein-assemblies and HLA-G-positive exosomes, and are they detected with conventional assays? These questions have not been actively addressed so far, and some investigators have indicated that due to the low abundance of G2 and G4-7 mRNA transcripts in the placenta, the physiological effects are provided essentially by HLA-G1 (89, 205). Conflicting with this notion is the immune regulatory capacity of the HLA-G5 isoform that, despite the fact that this transcript is scant in the placenta, has proven potent as an immunosupressor in several studies (59, 87). Another explanation for the lack of association between HLA-G genetics and preeclampsia could be due to different methodological approaches, small-scale studies on different ethnic populations, or explained by the fact that preeclampsia is a multifactorial disease that presents with different degrees of severity, and additionally, in an early- and late-onset form, possibly with distinct etiologies (206).

The involvement of HLA class Ib in preeclampsia remains controversial. The function of HLA-F is unknown, and despite findings showing that HLA-E is involved in immune suppression, soluble HLA-E levels seem not associated with preeclampsia. More studies, not only focusing on the two non-synonymous alleles classically investigated, are needed. The functional significance of HLA-G in pregnancy is more complex than HLA-E and -F. However, the high expression of HLA-G compared to HLA-E and -F in the placenta, and the presence of HLA-G in semen, the endometrium, in the matured cumulus–oocyte complex, as well as the rise in soluble level after conception imply an important role for HLA-G in early pregnancy (42, 45). Furthermore, the dual role of HLA-G in immune regulation and spiral artery remodeling underscores its importance and multifaceted activities. So far, aberrant HLA-G expression is a likely contribution to preeclampsia. As isoform-specific functions are possible to exist, more studies on this are highly warranted.

The etiology of preeclampsia is multifactorial and involves interactions between immune cells and HLA class Ib molecules, possibly as early as during conception or embryogenesis (46). And since an interaction in essence is a mutual or reciprocal action or influence, any one unfavorable genetic or immunological contribution either from the mother, the father, or the fetus, may tip the steady-state immune balance in a direction unfavorable for pregnancy – consequently leading to preeclampsia. Further in-depth investigation will help to elucidate the precise mechanism of HLA class Ib receptor recognition and signaling, and the role of these interactions in successful reproduction.

## Conflict of Interest Statement

The authors declare that the research was conducted in the absence of any commercial or financial relationships that could be construed as a potential conflict of interest.
